# Effects of Excess Manganese on the Xylem Sap Protein Profile of Tomato (*Solanum lycopersicum*) as Revealed by Shotgun Proteomic Analysis

**DOI:** 10.3390/ijms21228863

**Published:** 2020-11-23

**Authors:** Laura Ceballos-Laita, Elain Gutierrez-Carbonell, Daisuke Takahashi, Andrew Lonsdale, Anunciación Abadía, Monika S. Doblin, Antony Bacic, Matsuo Uemura, Javier Abadía, Ana Flor López-Millán

**Affiliations:** 1 Plant Stress Physiology Group, Plant Nutrition Department, Aula Dei Experimental Station, CSIC, P.O. Box 13034, 50080 Zaragoza, Spain; ceballos.laita@gmail.com (L.C.-L.); Elain.Gutierrez@sciex.com (E.G.-C.); mabadia@eead.csic.es (A.A.); anaflorlopez@gmail.com (A.F.L.-M.); 2 United Graduate School of Agricultural Sciences, Iwate University, Morioka 020-8550, Japan; dtakahashi@mail.saitama-u.ac.jp (D.T.); uemura@iwate-u.ac.jp (M.U.); 3 School of Biosciences, The University of Melbourne, Parkville, VIC 3052, Australia; a.lonsdale@student.unimelb.edu.au; 4 La Trobe Institute for Agriculture & Food, Department of Animal, Plant & Soil Sciences, AgriBio Building, La Trobe University, Bundoora, VIC 3086, Australia; M.Doblin@latrobe.edu.au (M.S.D.); t.bacic@latrobe.edu.au (A.B.); 5 Department of Plant-bioscience, Faculty of Agriculture, Iwate University, Morioka 020-8550, Japan

**Keywords:** xylem sap, manganese toxicity, proteome, tomato, shotgun proteomics

## Abstract

Metal toxicity is a common problem in crop species worldwide. Some metals are naturally toxic, whereas others such as manganese (Mn) are essential micro-nutrients for plant growth but can become toxic when in excess. Changes in the composition of the xylem sap, which is the main pathway for ion transport within the plant, is therefore vital to understanding the plant’s response(s) to metal toxicity. In this study we have assessed the effects of exposure of tomato roots to excess Mn on the protein profile of the xylem sap, using a shotgun proteomics approach. Plants were grown in nutrient solution using 4.6 and 300 µM MnCl_2_ as control and excess Mn treatments, respectively. This approach yielded 668 proteins reliably identified and quantified. Excess Mn caused statistically significant (at *p* ≤ 0.05) and biologically relevant changes in relative abundance (≥2-fold increases or ≥50% decreases) in 322 proteins, with 82% of them predicted to be secretory using three different prediction tools, with more decreasing than increasing (181 and 82, respectively), suggesting that this metal stress causes an overall deactivation of metabolic pathways. Processes most affected by excess Mn were in the oxido-reductase, polysaccharide and protein metabolism classes. Excess Mn induced changes in hydrolases and peroxidases involved in cell wall degradation and lignin formation, respectively, consistent with the existence of alterations in the cell wall. Protein turnover was also affected, as indicated by the decrease in proteolytic enzymes and protein synthesis-related proteins. Excess Mn modified the redox environment of the xylem sap, with changes in the abundance of oxido-reductase and defense protein classes indicating a stress scenario. Finally, results indicate that excess Mn decreased the amounts of proteins associated with several signaling pathways, including fasciclin-like arabinogalactan-proteins and lipids, as well as proteases, which may be involved in the release of signaling peptides and protein maturation. The comparison of the proteins changing in abundance in xylem sap and roots indicate the existence of tissue-specific and systemic responses to excess Mn. Data are available via ProteomeXchange with identifier PXD021973.

## 1. Introduction

Manganese (Mn) is an essential micronutrient that plays important roles in many plant physiological processes [1]. Manganese is required for photosynthesis as a constituent of the O_2_ evolving complex in photosystem II, and acts as a cofactor of multiple enzymes in metabolic pathways such as the Krebs cycle and in redox homeostasis [2,3,4]. However, when in excess, Mn can be toxic to plants [3]. Manganese occurs in soils largely in oxide forms, and is taken up by plants as Mn^2+^, with its availability being influenced by soil parameters such as soil structure and composition, water content, microbial activity and especially pH. Plant-available Mn can increase considerably in acidic soils, and this may lead to Mn toxicity [1,5]. Increases in soil acidity are driven by the use of intensive horticulture and acidifying fertilizers [3,6,7], as well as by climate events such as elevated ozone levels, increased ambient temperatures and emission of acidic gases [8,9,10].

Excess Mn concentrations in roots and shoots of plants leads to leaf chlorosis and stunted growth, increases in the concentration of reactive oxygen species (ROS), and consequently oxidative stress, and perturbations in carbohydrate, amino acid, protein and nucleic acid metabolism [11,12,13,14,15,16]. Excess Mn also alters photosynthesis and photosynthetic pigments and hormone levels [17,18]. To cope with excess Mn, plants elicit several stress-response processes, including decreases in the translocation of this metal from roots to shoots [19,20,21] and regulation of the mechanisms involved in its acquisition and distribution inside the plant, including sequestration in the vacuole [22,23,24]. The plant vasculature plays an essential role in these homeostatic mechanisms, since it constitutes the main conduit for long distance transport, distribution and delivery of Mn.

The vascular system is composed by the phloem and xylem conduits, which transport a myriad of molecules, with the apoplast compartment acting as an intercellular space between them and also between cells [25,26]. The xylem is an essential component of this system, whose primary role is the transport from roots to shoots of water and nutrients, including Mn. The xylem sap flows through the treachery elements that compose the xylem vessels, being driven by the negative pressure created by transpiration and/or by the positive root pressure created by the differences in water potential between the soil and the root system [27]. Although the most abundant components of the xylem sap are water and minerals, it also contains many other compounds in low quantities, including carbohydrates, hormones, secondary metabolites, peptides and also proteins [28,29,30,31,32,33]. The protein profile of the xylem sap is unique, and includes many proteins containing N-terminal signal peptides. These proteins are synthesized in the root and subsequently loaded to the xylem sap via the so-called conventional secretory pathway [33,34,35,36]. However, not all proteins present in the xylem sap are secreted by this pathway. Other proteins without a signal peptide are thought to be secreted via different mechanisms; the pathway for proteins that are non-classically secreted is called unconventional protein secretion (UPS) [37]. Different software tools have been developed for predicting which proteins are likely to undergo UPS, first based on bacterial and mammalian protein data [38], whose applicability to plant systems is under debate [39] and, more recently, on plant protein data [40]. The presence of proteins originating from neighboring tissues has also been commonly reported in the xylem sap proteome, suggesting some degree of cytoplasmic contamination [33].

Proteomic approaches, either gel (2-DE) or non-gel-based, are powerful tools to understand the responses that plants elicit when facing metal stresses [41,42]. The xylem sap proteome is expected to reflect changes in plant metabolism upon excess Mn and could also be an essential key for understanding Mn homeostasis. Limited information is still available on the effects of excess Mn on the xylem sap proteome, with current findings being mainly restricted to whole root and leaf tissues. In roots, proteomic analyses on Mn toxicity have been carried out by 2-DE using two *Citrus* species [20] and *Glycine max* [21], and by shotgun proteomics and 2-DE using *Solanum lycopersicum* (tomato) [42]. These studies have suggested that excess Mn causes alterations in the structure and lignin composition of root cell walls and an impairment of metabolic pathways involved in energy production, which are probably responsible for the growth inhibition observed in the roots of plants grown in the presence of excess Mn [21,42]. Other alterations in roots include changes in protein turnover and an activation of plant defense and ROS protection mechanisms [21,43,44,45]. In leaves, proteomic studies have suggested that Mn toxicity causes decreases in CO_2_ fixation and large increases in ROS [20,44,46]. On the other hand, studies on xylem sap obtained from plants subjected to other biotic and abiotic stresses, including Mn deficiency, indicate that the proteins in xylem sap might play important roles in nutrient stress signaling [32,41,47,48,49].

In this study we have tested the hypothesis that excess Mn causes changes in the xylem sap protein profile of tomato, using a shotgun proteomics approach. Tomato was chosen as the model plant because its genome has been sequenced and the combination of root pressure and turgid stems allows for sufficient xylem sap collection by de-topping. Additionally, we have compared the changes in the protein profiles of the xylem sap and roots (the latter published in [42]), with the aim of finding changes that may constitute a systemic response in contrast with those specific to the roots or the xylem sap. Results support that excess Mn elicited an overall deactivation of metabolic pathways, with alterations in proteins associated with the cell wall, protein turnover the redox environment and signaling pathways.

## 2. Results

### 2.1. Xylem Sap Collection and Mineral Composition

Hydroponically grown tomato plants (26 days old) were treated with either 4.6 µM (control) or excess Mn (300 µM MnCl_2_). Plants grown in excess Mn showed toxicity symptoms 6 days after the treatment onset. Eight days after imposing the treatments, plants were de-topped, and the xylem sap was collected. Visual toxicity symptoms at sampling time included brown spots in stems and leaf veins, 50% chlorophyll decreases in young leaves, and increases in Mn concentrations of 17- and 21-fold in roots and leaves, respectively, whereas the concentrations of Fe, Zn and Cu were not affected [42]. Xylem bleeding rates were similar in plants treated with excess Mn and in the controls (0.52 and 0.56 mL g^−1^ DW h^−1^, respectively) whereas the protein concentration in the xylem was on average 3-fold higher in plants grown in the presence of high Mn compared to the controls (27 and 9 ng protein µL^−1^, respectively). In addition, leaf transpiration rates were reduced by 52% in the high Mn-grown plants when compared to the controls (10.9 and 5.2 mmol H_2_O m^−2^ s^−1^, respectively) (Table 1).

Manganese concentration in the xylem sap was 59-fold higher in the plants grown in excess Mn than in the controls (386 and 7 µM, respectively; Table 1), slightly higher than the concentration within the hydroponic solution in which they were grown. On the other hand, the concentrations of Fe and Zn were 67% and 16% lower, respectively, in the high Mn grown plants when compared to the controls, and no significant differences were found for Cu.

### 2.2. Proteins Detected, Class Annotation and PCA Analysis

The shotgun proteomics (LC-MS/MS) analysis detected 1783 proteins in tomato xylem sap, and 1230 were initially quantified by the Progenesis software (Table S1). Of these, 668 proteins were present in all six biological replicates of at least one treatment and reliably identified and quantified with at least 2 peptides. Only these protein species were considered in this study. The full list of proteins detected is shown in Table S1, and the list of the corresponding peptides is available in the ProteomeXchange Consortium via the Pride partner repository within the dataset identifier PXD021973. All identified proteins were annotated using GO and Uniprot (see Section 4.5) into one of ten different functional classes: polysaccharide related, oxido-reductases, protein metabolism, carbohydrate metabolism, lipid metabolism, signaling/regulation, defense, nutrient reservoir, unknown and a miscellaneous group containing categories not belonging to the previous groups.

**Figure 1:**
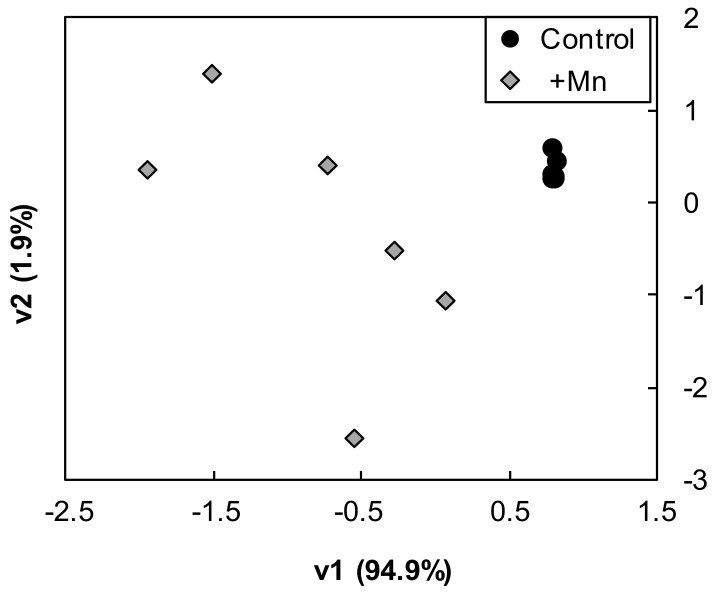
Score scatter PCA (Principal Component Analysis) plot of the Mn-excess treated samples when compared to the controls. PCA was carried out using SPSS Statistical software (v. 24.0) and included proteins showing statistically significant changes (ANOVA, *p* ≤ 0.05) when compared to the controls. Black dots and grey diamonds depict control and Mn-treated samples, respectively.

All proteins showing statistically significant changes (ANOVA; *p* ≤ 0.05) were used for a PCA analysis. This analysis showed a good separation between treatments, with the first and second components (PC1 and PC2, respectively) explaining approximately 94.9 and 1.9% of the variation, respectively (Figure 1; Table S2). Proteins with large positive PC1 scores (13 proteins) were from the protein metabolism, oxido-reductases, defense, lipid, carbohydrate and polysaccharide classes (3, 2, 2, 2, 1 and 1 proteins, respectively) and the miscellaneous group (2 proteins), whereas those with large negative contributions (16 proteins) were from the polysaccharide, defense, oxido-reductases and protein classes (8, 3, 3 and 2 proteins, respectively) (Table 2).

### 2.3. Effect of Excess Mn on the Xylem Sap Protein Profile

**Figure 2:**
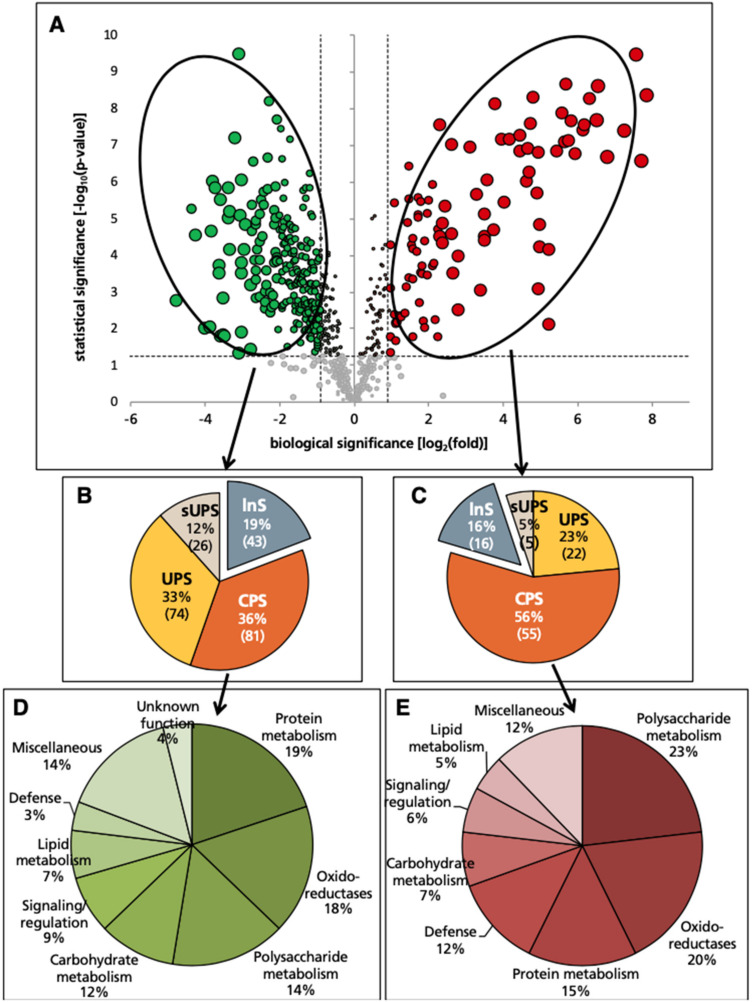
Effect of excess Mn on the xylem sap protein profile using label-free shotgun proteomic analyses. (**A**) Volcano scatter plot with the 668 identified and quantified proteins (peptides assigned to a protein and used for quantification ≥2). Proteins decreasing and increasing in relative abundance (ANOVA, *p* ≤ 0.05) are in green and red, respectively, whereas those whose relative abundance was unaffected are in grey. Light and dark colors are used for proteins meeting only the statistical threshold (ANOVA, *p* ≤ 0.05) and both the statistical and biological (>2-fold increase [98 proteins] or ≥50% decrease [224 proteins]) thresholds, respectively. The dot size is proportional to the fold-change. (**B**,**C**) Pie charts depicting the classification using different software tools (see Materials and Methods) for proteins showing decreases (**B**) and increases (**C**) in relative abundance. CPS: conventional secretory pathway; UPS: unconventional secretory pathway; sUPS: suggested unconventional secretory pathway; lnS: likely non-secretory. (**D**,**E**) Functional classification based on GO biological process and domain annotations of secretory (CPS + UPS + sUPS) proteins showing decreases (**D**) and increases (**E**) in relative abundance.

Excess Mn caused statistically significant (ANOVA, *p* ≤ 0.05) and biologically relevant (defined in this study as having ≥2-fold increases or ≥50% decreases) changes in 322 protein species in the xylem sap proteome. From these, 224 (70%) decreased in abundance (Table S3), whereas 98 (30%) increased in abundance (Table S4). Changes found are shown in a volcano plot (Figure 2A), which depicts the relationship between statistical [−log_10_(*p*-value)] and biological [log_2_(fold-change)] significances.

All these proteins were grouped into one of four categories using different software tools to distinguish secretory proteins from intracellular (cytoplasmic) contaminants. Those with a N-terminal leader signal sequence detected by SignalP, TargetP and/or SecretomeP were identified first and labelled conventional protein secretory (CPS). Proteins without a classical signal peptide (SP) that can be secreted through unconventional pathways were predicted in multiple ways. As pointed out previously, the accuracy of SecretomeP in predicting such proteins in plants is not optimal, being significantly less than observed for mammalian proteins [39], but this tool is still commonly used in plant studies. Thus, to complement this strategy, we used a new plant-based-tool, LSPpred [40], considered to be an improvement in UPS prediction in the absence of a well-defined plant unconventional secretory protein dataset. Those proteins lacking a SP but predicted to be secretory by at least one of the LSPpred or SPLpred modules of LSPpred were labelled as unconventional protein secretory (UPS). Proteins only predicted by the mammalian-based SecretomeP tool were assigned to the category of suggested unconventional secretory proteins (sUPS). A fourth group with proteins not predicted to be secretory by any prediction tool were labelled likely non-secretory (lnS) (Figure 2B,C; protein species are listed in Tables S3 and S4). A second volcano plot, with the distribution of the different categories, is shown in Figure S1.

As expected, the different tools used to predict unconventional secretory proteins gave contrasting results, with a total of 142 proteins being predicted to be so by at least one tool: 36 by LSPpred only, 5 by SPLpred only, 37 by LSPpred and Secretome P, 11 by SPLpred and Secretome P, 9 by LSPpred and SPLpred, 7 by all three algorithms and 37 only by the non-plant-based algorithm SecretomeP (Figure S2). Forty-four more proteins were considered as likely to be non-secretory because they were not selected by any of the algorithms (Tables S3 and S4). Among the proteins considered by the prediction tools to be secretory, 15 (5 by LSPpred only, 1 by SPLpred only, 1 by SPLpred and Secretome P, 2 by LSPpred and SPLpred and 6 only by SecretomeP) were judged to be contaminants from their clear intracellular localization (e.g., chloroplastic) and added to the lnS group (Tables S3 and S4), adjusting the final number of possible UPS proteins to 127 (96 UPS and 31 sUPS).

### 2.4. Protein Species Decreasing in Abundance

Among the 224 proteins decreasing in relative abundance (dark green dots in Figure 2A; Table S3), 181 (81%) were classified as secretory (including 81 CPS, 74 UPS and 26 sUPS; Figure 2B). The remaining 43 proteins were in the lnS group and were excluded from the biological interpretation, since they could originate from cytoplasmic contamination from neighboring cells. The most highly represented classes based on GO biological process in these secretory proteins (CPS + UPS + sUPS) were protein metabolism (18%), oxido-reductases (18%) and polysaccharide metabolism (14%), followed by carbohydrate (12%), signaling/regulation (9%), lipid metabolism (7%) and defense (3%) (Figure 2D). Decreases in abundance were generally moderate (83% of them ranging between 50 and 80%), with only 23 proteins, scattered among the different metabolic classes, decreasing more than 90% as a result of excess Mn (Table 3).

In the protein metabolism class, decreases in relative abundance were measured in 27 proteolysis-related proteins (18 CPS, 6 UPS and 3 sUPS) and five ribosome structural components (one of them UPS and four sUPS) (Table S3). Proteolytic proteins included four subtilisins and two serine endopeptidases (MEROPS family S8), three serine carboxy-peptidases (MEROPS family S10), two cysteine proteases and one glycine decarboxylase (MEROPS family C1), one aspartic peptidase and three aspartyl proteases (MEROPS family A1), one processing peptidase (MEROPS family M16), one lipid associated peptidase (MEROPS family C85), a neproxin (MEROPS family U74) and seven proteins related to proteasome [50]. The last protein in this group was a CPS (12g014270.2.1) involved in glycopeptide hydrolysis (all accession numbers mentioned in the text are Solyc from the tomato ITAG3.2 database).

In the oxidoreductase class (33 proteins: 17 CPS, 11 UPS and 5 sUPS; Table S3), the most represented families were peroxidases, Cu oxidases and aldehyde/histidinol dehydrogenases (seven proteins each). The twelve remaining proteins in this class included six miscellaneous reductases (10g081440.2.1, 02g087230.3.1, 01g108630.3.1, 06g062280.3.1, 06g083690.3.1 and 08g028690.3.1), a thioredoxin peroxidase (06g049080.3.1), a germin family member (01g102390.3.1), a carbonic anhydrase (04g080570.3.1) and three miscellaneous oxidases (10g005110.3.1, 08g068420.3.1 and 12g008640.2.1).

In the polysaccharide metabolism class (26 proteins: 21 CPS and 4 UPS and 1 sUPS; Table S3), most proteins (20) belong to several glycoside hydrolase (GH) subfamilies (GH 3, 5, 10, 16, 17, 19, 20, 27, 28, 32, 35, 79 and 127), with GH17 and GH 28 being the most highly represented (four and three proteins, respectively). The remaining six proteins in this group included two pectin acetyl esterases (10g038130.2.1 and 07g062210.3.1), a pectin methyl esterase inhibitor (PMEI; 11g005820.1.1), a rhamnogalacturonate lyase (04g076660.3.1) and two family 75 glycosyl transferases (GTs) (04g005340.2.1 and 05g012070).

In the carbohydrate class (22 proteins: 19 UPS and 3 sUPS; Table S3), there were six proteins involved in glycolysis (12g095760.2.1, 01g110360.3.1, 09g009260.3.1, 10g083570.2.1, 04g082630.3.1 and 04g009030.3.1), eight related to TCA (12g009400.2.1, 07g064800.3.1, 01g100360.3.1, 01g094200.3.1, 08g077920.3.1, 10g074500.2.1, 01g101040.3.1 and12g005080.2.1), two participating in the pentose phosphate shunt (05g010260.3.1 and 03g121720.2.1) and six involved in monosaccharide metabolism: two related to galactose (01g058390.3.1 and 05g024415.1.1), two related to glucose (02g088690.3.1 and 11g011960.2.1) and one each to rhamnose (08g080140.3.1), and xylose (11g066720.2.1).

In the signaling/regulation class (16 proteins: 9 CPS, 6 UPS and 1 sUPS; Table S3), five proteins were fasciclin-like arabinogalactan-proteins (FLAs), five more were involved in chaperone-mediated protein folding (four of them with a Cpn60 domain (02g063090.3.1, 05g056310.3.1, 06g065520.3.1 and 11g069000.2.1) and four were kinases (three of them with a leucine-rich repeat receptor: 01g086920.3.1, 10g050110.1.1 and 03g111670.3.1). The remaining two proteins were an abscisic acid receptor (08g076960.1.1) and an adenylyl cyclase-associated protein (10g051340.2.1).

In the lipid metabolism class (13 proteins: 7 CPS, 5 UPS and 1 sUPS; Table S3), eight were esterases and lipases, three were lipid-transfer proteins (03g079880.3.1, 03g093360.3.1 and 08g067500.1.1) and the remaining two were a fatty acid dehydratase (01g105060.3.1) and an acyl-transferase (01g006980.3.1).

Six more proteins were in the defense class (5 CPS and 1 UPS; Table S3), including three osmotins belonging to the pathogenesis-related (PR) family 5 (PR-5; 02g087520.3.1, 04g081550.3.1 and 05g053020.3.1), two stress related proteins (07g007760.3.1 and 03g096460.3.1) and a lectin family protein with a Ricin B-like domain (01g010750.3.1).

A miscellaneous class included 24 proteins (19 UPS and 5 sUPS; Table S3), with eleven involved in amino acid metabolism, and two each involved in purine metabolism, ATP hydrolysis coupled proton transport and protein transport, among others.

Finally, eight proteins (4 CPS, 3 UPS and 1 sUPS; Table S3) were classified as having an unknown function.

Marked decreases in abundance (>90%) were found in 23 proteins (Table 3): a β-galactosidase (GH35; 09g092170.2.1) and the trichome birefringence-like protein classified as a pectin acetyl esterase (07g062210.3.1) in the polysaccharide class; an aspartyl protease (from MEROPS family A1), a inducible plastid-lipid associated protein (07g064590.3.1 from MEROPS family C85) and two ribosome structural components in the protein metabolism class; an ascorbate peroxidase (06g005160.3.1), two cupredoxins (04g074740.3.1 and 08g066740.3.1) and a NAD(P)-binding Rossmann-fold protein with SDR domain (08g028690.3.1) in the oxido-reductase class; proteins related to TCA (01g094200.3.1 and 01g101040.3.1), galactose and glucose metabolism (01g058390.3.1 and 02g088690.3.1, respectively), in the carbohydrate metabolism class; two proteins with chaperone (Cpn60) activity (02g063090.3.1 and 05g056310.3.1) and two kinases with a leucine-rich repeat receptor (01g086920.3.1 and 10g050110.1.1) in the signaling/regulation class; a phospholipase in the lipid metabolism class (01g107990.3.1), three more proteins in the miscellaneous class and a remorin having an unknown function (06g035920.3.1). The most remarkable decreases were observed in the blue copper oxidase (04g074740.3.1), the NAD (P)-binding Rossmann-fold protein (08g028690.3.1) and the leucine-rich repeat receptor (01g086920.3.1), which were barely detectable under excess Mn (Table 3).

### 2.5. Protein Species Increasing in Abundance

Among the 98 proteins increasing in relative abundance (dark red dots in Figure 2A; Table S4), 82 (84%) were classified as secretory (including 55 CPS, 22 UPS and 5 sUPS; Figure 2C). The remaining 16 were in the lnS group and therefore were excluded from this analysis. The most represented classes in these secretory proteins (CPS + UPS + sUPS) were polysaccharide metabolism (23%) and oxido-reductases (20%), followed by protein metabolism (16%), defense (11%), signaling/regulation (9%) and carbohydrate and lipid processes (6% each) (Figure 2E). The three most highly represented classes were similar to those found for proteins decreasing in abundance; however, within a given class, proteins increasing and decreasing belonged to different subfamilies.

In the polysaccharide-related metabolic class (19 proteins, all CPS; Table S4), GHs were predominant (16 of 19 proteins), with the most abundant subfamilies being GH17, GH18 and GH19 (six, three and four proteins, respectively), and with the subfamilies GH5, GH16 and GH20 accounting for one protein each. The subfamily GH18 was not found among those decreasing in abundance upon excess Mn. The remaining three proteins in this group were a pectin acetyl esterase (08g005800.3.1), a polysaccharide lyase family 7 protein (11g005480.2.1) and an invertase inhibitor (12g099200.2.1).

In the oxido-reductase class (16 proteins: 10 CPS, 5 UPS and 1 sUPS; Table S4), most (88%) presented moderate increases between 2- to 14-fold. This class included eight peroxidases, two aldehyde/histidinol dehydrogenases and two thioredoxins, whereas Cu oxidases were absent. The four remaining oxido-reductases (3 UPS and 1 sUPS) were a monodehydroascorbate reductase (MDAR; 09g009390.3.1), a glutathione reductase (09g091840.3.1), a quinone reductase (02g079750.3.1) and a phenylacetaldehyde reductase (01g008550.4.1).

As for proteins with reduced abundance, most protein species in the protein metabolism class (13 proteins: 10 CPS, 2 UPS and 1 sUPS; Table S4) with increased abundance belong to proteolytic processes. These included proteins of the MEROPS subfamilies S8 (two), S9 (one) and A1 (three) and five protease inhibitors from the MEROPS families I3 and I13 (these two subfamilies and subfamily S9 were not among those decreasing in abundance). Finally, this group also included the UPS elongation factor (11g072190.2.1) and an alanine-tRNA ligase (03g097290.3.1) involved in protein synthesis.

In the defense class (9 proteins: 7 CPS and 2 UPS; Table S4), there were seven cysteine-rich secreted proteins (CRSPs), with either Barwin (01g097240.3.1 and 01g097270.3.1), allergen V5 (00g174340.2.1 and 01g106620.2.1), MLP (07g005380.3.1 and 09g090980.3.1; both UPS) or Gnk2 (12g005720.1.1) domains (Table S4). The remaining two proteins were an osmotin of the PR-5 family (08g080585.1.1), and a stress up-regulated Nod 19 protein 12g042380.2.1).

Other metabolic classes accounted for one third of the total proteins increasing in abundance: signaling/regulation (four protein folding related proteins, two kinases and a nuclease; 5 CPS and 2 UPS); carbohydrate metabolism (two proteins from the glycolytic pathway, two from the pentose phosphate shunt and one involved in xylose metabolism; 1 CPS and 4 UPS); lipid metabolism (two carboxylesterases, an esterase/lipase, an enoyl reductase and a thiolase; 1 CPS, 3 UPS and 1 sUPS); amino acid metabolism (four amino transferases, a cysteine synthase and a kynurenine formidase; 1 CPS, 4 UPS and 1 sUPS), one CPS acid phosphatase and one sUPS adenylate kinase (Table S4).

Marked increases in abundance (≥25-fold) were found in 28 proteins (Table 3): ten glycosyl hydrolases (from GH families 17, 18 and 19) and a citrate binding protein with an alginate lyase domain (11g005480.2.1) in the polysaccharide class; two subtilisin-like proteases (MEROPS S8) (08g079870.3.1 and 08g079900.3.1), the tolB protein (06g008620.1.1, MEROPS S9) and five protease inhibitors from the MEROPS families I3 and I13 in the protein metabolism class; the peroxidases 03g006700.3.1 and 04g071890.3.1 in the oxido-reductase class, and five PR proteins in the defense class. The most remarkable increases were observed in the CPS β-1,3-glucanases 01g008620.3.1 and 01g059965.1.1 (234- and 189-fold, respectively), the MEROPS family I3 protease inhibitor 03g098740.1.1 (209-fold) and the PR protein 01g097240.3.1 (154-fold) (Table 3).

Thirteen more secretory proteins (11 CPS and 2 UPS) had changes in relative abundance between 8.3- and 25-fold, and therefore will have increased ≥25-fold if the basis used for comparison was total protein concentration in the xylem sap (that increases 3-fold with excess Mn) instead of relative abundance (Table S5). These proteins belong to the oxido-reductases, defense, signaling-regulation, polysaccharide metabolism and lipid metabolism classes (3, 3, 2, 1 and 1 proteins, respectively), with 3 more being included in the amino acid metabolism class.

### 2.6. Comparison of Changes Observed in the Xylem and Root Protein Profiles in Response to Excess Mn

**Figure 3:**
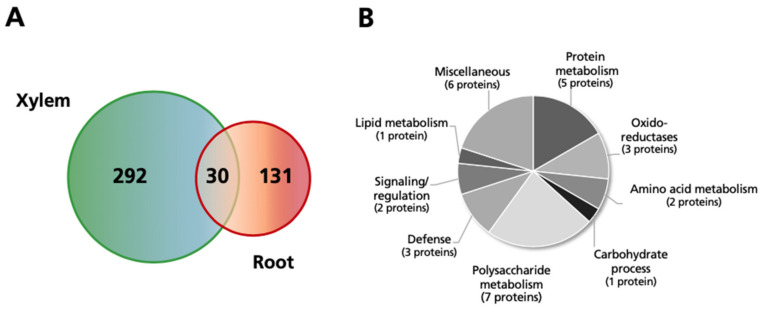
(**A**) Venn diagram comparing the number of proteins changing as a result of excess Mn in roots and xylem sap of tomato plants (ANOVA, *p* ≤ 0.05), identified and quantified with at least two peptides, and above the threshold level (fold change ≥2 or ≤0.5). (**B**) Functional classification of the common proteins (30) showing changes in xylem and root samples of plants grown with excess Mn.

The changes in the tomato xylem sap protein profiles as a result of excess Mn were compared with those observed in the root protein profiles of tomato grown in the same conditions, which were assessed using a combined 2-DE and shotgun proteomics approach [42] (Figure 3). This comparison yielded 30 proteins (13 CPS, 10 UPS, 2 sUPS and 5 lnS) in common between xylem sap and roots, with most (28) following the same trends in both tissues as a result of excess Mn (16 and 12 proteins decreasing and increasing in abundance in both tissues, respectively). The only proteins changing trends were a CPS GDSL esterase/lipase (12g017460.1.1), which increased 14-fold in the xylem sap and decreased by 50% in roots, and the UPS heat shock protein 70 (01g106210.3.1), which increased 3.4-fold in xylem sap but decreased 60% in roots (Table 4).

Many of the proteins decreasing in abundance in both tissues were in the UPS and sUPS categories (69%; 9 and 2 proteins, respectively), whereas most of those increasing (83%; 10 proteins) were in the CPS category (Table 4). The magnitude of changes was similar for protein species decreasing in abundance in the roots and xylem sap, whereas increases in abundance were, generally speaking, more marked in the xylem than in roots. Secretory proteins increasing in abundance in both tissues were classified mainly in four metabolic classes (polysaccharide metabolism, proteolysis, oxido-reductases and defense) and included five GHs, a peroxidase, a Kunitz-type protease inhibitor and three PR proteins, whereas those decreasing in abundance were scattered among different metabolic classes, with protein metabolism being the most represented, with four proteins.

## 3. Discussion

Excess Mn caused large Mn concentration increases in the xylem sap when compared to those reported in the roots and leaves of plants grown in the same system [42], indicating the presence of an active translocation of this metal from roots to shoots. Such a system has been proposed to be related to the relative tolerance to this stress in some plant species [19,51].

The proteomic approach used in this study has allowed for the reliable identification and quantification of a large set of protein species (668) in xylem sap, with 82% (263 out of 322) of the proteins changing significantly being classified as secretory using several prediction tools (CPS + UPS + sUPS). A total of 181 and 82 secretory proteins showed decreases and increases in relative abundance, respectively, suggesting a relative deactivation of metabolic pathways upon this nutritional stress.

Our data suggest that 18% of the proteins identified and quantified (59) in this study may have an intracellular origin and thus reflect cytoplasmic and organelle contamination. The percentage of secretory proteins found in the present study (82%) falls within the range of those reported (70–90%) using a similar shotgun approach in xylem sap of several plant species [36,41,49]. Furthermore, the separation between samples was largely unaffected by contamination, since only two proteins contributing significantly to component 1 in the PCA analysis were lnS. However, total cytoplasmic/organelle contamination may be up to 28% if the 31 proteins (12% of the total secretory; classified as sUPS) predicted to be secreted via alternative secretion pathways by SecretomeP alone, a tool not designed for and displaying suboptimal performance with plant proteins [39,40], are included. Alternatively, some proteins not predicted as secretory by the algorithms can still be secreted to the xylem by still unknown mechanisms.

On the other hand, the use of plant-specific algorithms to identify secretory proteins used in this study detected 49 new proteins not found by SecretomeP, therefore highlighting the limitations of the use of a non-plant-based software in plant research.

### 3.1. Excess Mn Affects the Metabolism of the Cell Wall

When considering the changes in the secretory (CPS + UPS + sUPS) xylem sap protein profile upon excess Mn, polysaccharide metabolism was among the most represented categories, accounting for 17% of the total protein abundance changes (45 proteins; 26 decreasing and 19 increasing). This indicates that excess Mn caused significant alterations in the xylem sap cell wall, since most of these enzymes are extracellular and their function is to hydrolyze and/or rearrange glycoside bonds [52]. Most of these proteins were GHs (36), similar to previous xylem sap proteomic studies [41]. Protein decreases measured in GHs were relatively moderate, whereas GH increases were among the highest measured in this study (up to 234-fold). This suggests that even though the number of GH proteins increasing or decreasing in abundance was fairly similar, those increasing may play a more relevant role in the excess Mn response, leading to an overall degradation of the cell wall. Proteins in the polysaccharide category contributed to a large extent to the separation of treatments (nine proteins; Table 2), with five of them being glucan-endo-glucosidases (including 01g008620.3.1, one of the largest increases found), supporting the importance of the endo-hydrolyzing enzymes in the response to excess Mn. Similar increases in glucan-endo-glucosidases have been described in roots of tomato plants exposed to excess Mn [42]. Two GH subfamilies (GH 5 and 17 [53]) containing endo-glucanases, mainly acting in primary cell wall degradation, had different members increasing or decreasing as a result of excess Mn. However, other GH families had a distinct behavior. On one hand, subfamilies presenting mostly increases in abundance were GH 18 (three proteins) and GH 19 (four out of five), which are N-acetyl-hexosaminidases mainly involved in N-glycan degradation [54]. Therefore, in addition to cell wall degradation, the large increases in those proteins indicate a decrease in N-glycosylation, which is assumed to be a major post-translational modification of secreted proteins.

On the other hand, GH subfamilies with members having only decreases in abundance were glycosylases with very diverse catalytic and substrate specificities, acting in the degradation of xylans (xylosidases, GH3; endoxylanases GH10), mannans (GH5), galactosides (GH 27, GH28 and GH 35), β- glucuronidases (GH 79) and arabinofuranosides (GH127) [54]. Overall, these decreases, although quantitatively less important than the increases in other GHs, suggest that certain processes involving cell wall degradation may be reduced in the xylem sap of plants exposed to excess Mn, consequently leading to modifications to the cell wall. A similar situation has been described in the xylem sap of Mn-deficient plants [41]. These complex changes in GHs, in conjunction with the abundance changes measured in pectin acyl esterases (one increasing and two decreasing) and pectin methyl esterase inhibitors (one increasing and another decreasing), support that excess Mn likely causes an increased degradation of the primary cell wall, which occurs along with a re-arrangement of cell wall glycosides. A disorganization of the xylem vessels was observed using optical microscopy in *Glycine max* affected by Mn toxicity [55]. The fact that the protein concentration in the xylem sap of plants affected by excess Mn was 3-fold higher than in the controls, whereas the xylem flow rates were similar, may be in line with an increased cell wall permeability in these plants. It should be noted that since GHs can have effects in primary, secondary and/or both cell walls, functional studies are needed to confirm these hypotheses.

A significant proportion (19%; 49) of proteins changing in relative abundance in xylem sap with excess Mn were found in the oxido-reductase category, and some of these changes could be associated to Mn-induced modifications in the secondary cell wall. Among them, 13 proteins were secretory peroxidases (seven decreasing and eight increasing). Those increasing were orthologues of Prx 12, 25, 52, 53 and 73 from *Arabidopsis*, which are known to be involved in lignin formation and play a key role in controlling its deposition in the vascular tissue [56,57,58,59,60,61]. However, among the seven peroxidases with decreased abundance, five have been described as stress-response proteins [62,63,64]. These results indicate that excess Mn also affects the secondary cell wall via peroxidases and reveals the complexity of the observed protein changes, since some lignin-related isoforms increased and some decreased. Peroxidases increasing in abundance may also have a ROS-protecting role by depleting H_2_O_2_ and therefore diminishing lipid peroxidation and protein oxidation [61,65]. Increased peroxidase activity has also been described to modulate Mn oxidation and compartmentalization, and thus affect Mn tolerance, in the leaf apoplast of *Vigna unguiculata* [66].

An alteration of lignin deposition and composition in the secondary cell wall is also supported by the decreases measured in seven Cu-oxidases, including six blue-Cu proteins with cupredoxin and phytocyanin domains, and the multi-Cu oxidoreductase laccase 3-like 06g082260.3.1. Increases in blue-Cu and multi-Cu proteins have been related to increases in lignification [67,68,69] and therefore, decreases in these proteins may lead to reduced lignin deposition, therefore affecting the cell wall structure.

### 3.2. Excess Mn Alters Protein Turnover

Approximately 17% of the secretory proteins affected by excess Mn were related to protein metabolism (46 proteins), and their changes suggest a decrease in proteolysis in the xylem sap. Nineteen of those decreasing in abundance were MEROPS proteolytic enzymes [50,70] and seven more were related to the proteasome 26S protein degradation pathway, indicating that both proteolytic pathways are affected by excess Mn. In agreement with these results, five proteins from the MEROPS I3/13 families involved in proteolysis inhibition presented substantial increases (between 31- and 210-fold). The deactivation of proteolytic processes is in line with the high protein concentrations measured in the xylem sap of plants grown with high Mn. In addition, excess Mn caused decreases in six ribosomal structural components (1 UPS and 5 sUPS), suggesting that protein synthesis might be compromised. These decreases would also be consistent with the large number of proteins decreasing in abundance in xylem sap as a result of excess Mn. Similar results have been described in the root proteome of tomato plants subjected to excess Mn, and it was hypothesized that decreased protein synthesis might be balanced with a less proteolytic environment, with this altered protein turnover leading to an accumulation of damaged proteins that could be responsible for some of the deleterious effects of Mn toxicity [42].

However, three proteases (two MEROPS S8B and one MEROPS S9) presented large increases (31- to 74-fold) in relative abundance with excess Mn, with the two MEROPS S8B contributing to a large extent to the separation of the treatments in the PCA analysis (Table 2). Moreover, three aspartic peptidases A1 also presented moderated increases (2- to 5-fold) in relative abundance, indicating that specific proteolytic events are induced upon excess Mn. These increases might play roles in either the degradation of ROS-damaged proteins as is the case under other nutritional stresses [71,72], in the maturation of cell wall proteins or in signaling systems mediated by peptide elicitors [73,74]. An alteration in protein turnover can also be inferred from the increases in three HSP70 chaperones and the decreases in four Cpn60 chaperones involved in protein folding.

### 3.3. Excess Mn Alters Defense Mechanisms

As discussed above, changes observed in the oxido-reductase category (16 proteins increasing and 33 decreasing) can alter the redox environment of the xylem sap and could potentially lead to oxidative stress in combination with the high Mn concentrations. Proteins increasing in abundance included several with antioxidant activity, such as two thioredoxins, a MDAR, as well as glutathione, quinone and phenylacetaldehyde reductases. Thioredoxins act as antioxidants by reducing proteins with disulfide bridges and participate in redox signaling [75], whereas MDAR is active in the ascorbate-glutathione cycle that detoxifies H_2_O_2_. In addition, peroxidases showing increases in abundance can also have a role protecting from excess Mn by removing H_2_O_2_, although in this process they would generate ROS. Indeed, ROS produced by extracellular peroxidases have been proposed to play a role in the IAA signaling pathway [76]. Increases in MDAR have also been previously described in roots of *C. sinensis* [20] upon Mn toxicity, although a decrease in the ascorbate-glutathione cycle was described in roots of *S. lycopersicum*, suggesting that the response may depend on the plant species [42]. It is worth mentioning that a decreased abundance was measured in a Mn-binding protein identified as CPS germin protein containing a cupin domain (01g102390.3.1) and in a thioredoxin peroxidase 1 (06g049080.3.1) with MnSOD activity.

In addition to oxidative stress-related proteins, 15 defense-related proteins (nine increasing, mostly cysteine-rich proteins with as yet unknown function, and six decreasing) were affected by excess Mn, with five of them having >25-fold increases in abundance. These changes indicate that excess Mn elicits a high level of stress that affects the xylem sap proteome. Increases in proteins of this type have previously been observed in *Nicotiana tabacum* [77] and *V. unguiculata* [44]. Two of the proteins with increased abundance (01g097240.3.1 and 01g097270.3.1) contain RlpA-like domains involved in lytic transglycosylase activity (InterPro entry), suggesting a role in cell wall modification, whereas two others (00g174340.2.1 and 01g106620.2.1) contain Tpx1 and CAP domains, described to regulate ion channel activity [78,79]. The latter may suggest an involvement in Mn homeostasis, perhaps to sequester Mn in the xylem sap, which was very high (386 µM) in plants grown in excess Mn. A protein in the defense category (09g090980.3.1; 26-fold increase) and an oxido-reductase (06g076630.3.1; 70% decrease) had large positive contributions to the separation of the treatments in the PCA analysis, again supporting the existence of a high stress environment affecting the xylem sap proteome of plants grown in excess Mn. On the other hand, large increases in the PR-10 (07g005380.3.1) and the major allergen (09g090980.3.1) proteins in the defense category, both containing a lipid-binding domain (START) that is found in signaling proteins, suggests the existence of a lipid-based signaling system (see discussion below).

### 3.4. Excess Mn Effects on Signaling and Regulation Mechanisms

Decreases in relative abundance of proteins within the signaling/regulation category suggest that several regulatory and signaling mechanisms may be repressed under high Mn. Among the 16 proteins decreasing in this category, one third (five proteins) are FLAs that belong to a subclass of arabinogalactan-proteins (AGPs), which participate in cell-adhesion and are involved in the regulation of stem development and response to abiotic stress [80,81]. These proteins affect stem biomechanics (strength and elasticity) by altering cellulose deposition in the stem secondary cell wall [81,82,83,84], and therefore their decreases are in line with the changes in peroxidases and Cu-oxidases affecting the secondary cell wall. Similar decreases in FLAs and Cu-oxidases were observed in the xylem sap of Mn-deficient plants [41], suggesting that alterations in Mn homeostasis may have a marked impact in stem biomechanics via FLAs.

Relative protein abundance changes were also observed in seven receptor proteins, including six leucine rich repeat receptor-like kinases (LRR; four decreasing 01g086920.3.1, 10g050110.1.1, 12g055720.2.1 and 03g111670.3.1 and two increasing 01g107670.2.1 and 10g052880.1.1) and a decrease in the PYL1 abscisic acid receptor 08g076960.1.1. The *Arabidopsis* orthologue (SRF6; At1g53730) of the LRR receptor 12g055720.2.1 participates in stress-related processes and probably in the positive regulation of leaf size [85], and therefore its decrease would be in line with the small leaf size of plants grown with Mn toxicity. The PYL1 receptor is required for ABA mediated responses such as stomatal closure [86,87,88], and therefore its decrease may imply an inhibition of the ABA signaling pathway. This category also contained changes in eight chaperones (three increasing and five decreasing) involved in protein folding and an increase in one protein folding activator (09g057670.3.1), whose changes are in line with the proposed alteration in the protein turnover.

Results also suggest the possible existence of a lipid-based signaling system as has already been proposed to exist in the xylem sap of Mn-deficient *S. lycopersicum* [41], as well as in the phloem sap of other species [89,90]. Changes in the abundance of lipases and lipid transfer proteins have been found in the xylem sap of plants growing with other nutritional deficiencies [32,41]. Excess Mn caused decreases in eight proteins with either lipase, esterase or phospholipase activities, including the PI-PLC X domain-containing lipase 01g107990.3.1, which shows one of the largest relative protein abundance decreases measured (Table 3). However, two more lipases increased, the UPS pepper-esterase-like 02g069800.1.1 and the GDSL esterase/lipase 12g017460.1.1. The large number of lipases decreasing in abundance would suggest a reduction of lipid catabolism, but since lipases and lipid-transfer proteins play important roles in signal transduction [91,92], these changes may also indicate the existence of a lipid-based signal transduction pathway which may be repressed or induced depending on the specific lipase acting in the signaling cascade. Furthermore, decreases in three lipid transfer proteins (03g079880.3.1, 08g067500.1.1 and 01g006980.3.1) and the increases in proteins containing the START lipid-binding domain (07g005380.3.1 and 09g090980.3.1) mentioned above also support that lipid metabolism plays a role in the responses to excess Mn.

Finally, the existence of a redox-based signaling mechanism can be supported by the large percentage of differential proteins belonging to the oxido-reductase category (18%), as it has been proposed to occur in other stress situations [93].

### 3.5. Excess Mn Affects General Metabolism

Excess Mn affected a significant number of carbohydrate metabolism-related proteins (27 in total), with most of them predicted to be UPS (23) or sUPS (2). Nevertheless, overall changes indicate a decrease in the TCA cycle (eight TCA proteins decreasing) which might imply a decrease in the production of reducing power and energy which has also been observed in roots of *S. lycopersicum* plants exposed to excess Mn [42]. The decreases in malate and isocitrate dehydrogenases could also potentially lead to an accumulation of malate, which has been reported to chelate Mn(II) in Mn-hyperaccumulating species [94,95]. Other energy-related pathways, including glycolysis (six proteins decreasing and two increasing) and the pentose phosphate shunt (two decreasing and two increasing), were also affected by excess Mn, indicating the complexity of the metabolic regulation in this stress.

Two proteins in the carbohydrate-related category, a UDP-glucose dehydrogenase (02g088690.3.1) and a galactokinase (01g058390.3.1) that participates in galactose catabolism, displayed remarkable decreases in relative abundance (>90%). UDP-glucose dehydrogenases are glucosyl donors that play an important role in C partitioning between sucrose synthesis and cell wall formation [96,97], and therefore this decrease also suggests the occurrence of excess Mn-induced changes in cell wall biosynthesis. Decreases in UDP-glucose dehydrogenases have also been observed in roots of *S. lycopersicum* plants grown with excess Mn [42].

Finally, as commented above, excess Mn also affected a large number of lipid-related proteins (18), with most of the changes (nine) being decreases in lipases, suggesting a decrease in lipid catabolism.

### 3.6. Comparison of Changes Induced by Excess Mn in the Root and Xylem Sap Proteomes

The list of xylem sap proteins identified in this study was compared to the root sap proteome generated in a previous study with *S. lycopersicum* plants grown in excess Mn conditions [42] to identify any commonalities. Indeed, 30 proteins were observed to be shared between the two proteomes, accounting for 19 and 9% of the proteins changing in relative abundance in the roots and in the xylem sap, respectively. This indicates that the majority of changes (81 and 91%, respectively) are specific to the roots or the xylem sap. However, a significant number of proteins change in abundance in both samples, with most (28 out of 30) following the same response upon excess Mn, suggesting a systemic response. As expected, most of the common proteins (25) were secretory, suggesting first that the contamination of the xylem sap is relatively low, and second that excess Mn induces changes in the extracellular space/cell wall in both tissues, with changes mostly following the same trend as commented above. Of the 16 common proteins decreasing in abundance in both proteomes 75% were UPS + sUPS (11; Table 4). These proteins have a diverse array of metabolic functions, suggesting that Mn toxicity causes a deactivation of a wide range of metabolic responses in both tissues via common players.

Most of the common proteins increasing in abundance in the root and xylem sap proteomes as a result of excess Mn were CPS secretory (10 out of 12), and distributed in four main metabolic classes, including polysaccharide metabolism, protein metabolism, defense and oxido-reductases (Table 4). Results indicate that these proteins most likely participate in extracellular processes elicited by excess Mn that may be part of overall specific responses. Proteins increasing in the polysaccharide and oxido-reductase classes were a 1,3-glucanase, four chitinases and a suberization-associated peroxidase that may be involved in cell wall modifications occurring upon excess Mn in both xylem sap and roots, whereas common proteins in the defense category included three pathogenesis related proteins. One of these, the lnS major allergen d 1 (09g091000.3.1), showed increases amongst the highest measured in both proteomic studies. This is a PR10 protein containing a hydrophobic pocket able to bind hormones and siderophores [98], which could be involved in hormone signaling or even Mn sequestration, and therefore constitutes a good candidate for future studies.

Finally, it is worth mentioning that upon treatment with excess Mn, proteins decreasing in relative abundance in both tissues were mainly non-classical secretory (UPS + sUPS), whereas those increasing were CPS. The implications of these findings would deserve future studies.

## 4. Materials and Methods

### 4.1. Plant Material and Sampling

Tomato (*Solanum lycopersicum*, cv. Tres Cantos) plants were grown in hydroponics in a controlled environment chamber (Fitoclima 10.000 EHHF, Aralab, Albarraque, Portugal) with a photosynthetic photon flux density (PPFD) of 400 µmol m^−2^ s^−1^ photosynthetically active radiation at leaf level, 80% relative humidity and a photoperiod of 16 h, 23 °C/8 h, 18 °C day/night regime. Seeds were germinated in vermiculite for 13 days in half-strength Hoagland nutrient solution containing 4.6 µM MnCl_2_. Seedlings were then transplanted to 10 L plastic buckets (16–18 plants per bucket) containing half-strength Hoagland nutrient solution and grown for an additional 13-day period. After this time, solutions were renewed, and control (4.6 µM MnCl_2_) and high Mn (300 µM MnCl_2_) treatments were imposed. The same treatments were used before to obtain root proteome profiles [42]. Xylem sap was collected eight days after treatment onset. Plants were de-topped approximately five mm above the mesocotyl using a carbon steel disposable scalpel (Nahita, Beriain, Spain) and the exuded fluid was collected from the cut surface. The sap collected during the first five min was discarded to minimize contamination with other plant fluids and broken cells, and the xylem sap sample was collected for 30 min using a micropipette tip. Samples were kept on ice during the entire collection period and proteins immediately precipitated. Samples were then stored at −80 °C until proteomic analysis.

### 4.2. Experimental Design

The experiment was repeated six times with independent batches of plants. Each batch of plants consisted of one 10 L bucket per treatment with 16–18 plants per bucket. In each batch of plants, xylem sap fluid from all plants in a given treatment was pooled together and considered as a biological replicate. Therefore, six biological replicates (n = 6) were used for LC-MS/MS, with all of them being used for protein identification and quantification.

### 4.3. Mineral Analysis

Micronutrients (Fe, Mn, Cu and Zn) in xylem sap were measured in four of the six biological replicates obtained as mentioned above (n = 4). The concentrations of micronutrients in the collected fluid were determined by ICP-MS (Inductively Coupled Plasma Mass Spectrometry; model Agilent 7500ce; Agilent Technologies, Tokyo, Japan) after digestion with 1% HNO_3_ (TraceSELECT Ultra, Sigma-Aldrich, Madrid, Spain), using mono-elemental standard solutions for ICP-MS (Inorganic Ventures, Christiansburg, VA, USA). Recovery and limit of detection were 95.8% and 2 μg Mn L^−1^, respectively.

### 4.4. Protein Extraction

Xylem sap proteins were solubilized as described in detail in [99,100]. Protein was quantified in diluted samples (Bradford kit, Sigma-Aldrich, St. Louis, MO, USA) using a microtiter plate spectrophotometer (Asys UVM 340, Biochrom Ltd., Cambridge, UK) and bovine serum albumin (Sigma) as standard.

### 4.5. Label Free Liquid Chromatography-Tandem Mass Spectrometry (LC-MS/MS)

Sample preparation for label free LC-MS/MS shotgun analysis was carried out as described previously [99,100]. Briefly, 5 µg of total proteins were subjected to 1-DE (for 10–15 min, in a precast Laemmli gel PAGEL NPU-10L, ATTO Corporation, Tokyo, Japan) to remove non-protein compounds, and the resulting gel band was cut into six pieces and pooled in a microtube. Proteins were in gel digested with trypsin and peptides were extracted subsequently.

Peptide separation was performed in an ADVANCE UHPLC system (Michrom Bioresources, Auburn, CA, USA) as described in [41]. Mass spectrometry analysis was carried out on an LTQ Orbitrap XL device (Thermo Fisher Scientific, Waltham, MA, USA), carrying out peptide ionization with a spray voltage of 1.8 kV and an ADVANCE spray source (Michrom Bioresources). Data acquisition parameters were set as in [101], and Xcalibur v. 2.0.7 (Thermo Fisher Scientific) was used as instrument control software.

Mass data analysis was performed as described previously [41,42,100,101,102]. Protein identification was carried out using the full peptide list with the Mascot search engine (version 2.4.1, Matrix Science, London, UK) and ITAG3.2 database (35,768 sequences; 11,956,401 residues). Search parameters were peptide mass tolerance ± 5 ppm, MS/MS tolerance ± 0.6 Da, one allowed missed cleavage, allowed fixed modification carbamidomethylation (Cys) and variable modification oxidation (Met), with peptide charges being set to +1, +2 and +3. Positive protein identification was assigned with at least two unique top-ranking peptides with scores above the threshold level (*p* < 0.05). Protein data were exported from Mascot .xml format and imported to Progenesis QI proteomics software (v. 2.0, Nonlinear Dynamics, Newcastle upon Tyne, UK), which then associates peptide and protein information. The mass spectrometry proteomics data have been deposited to the ProteomeXchange Consortium via the PRIDE [103] partner repository with the dataset identifier PXD021973 (in this dataset, samples 1–6 are for controls and 19–24 for plants grown with excess Mn).

Relative quantification of proteins was carried out using non-conflicting peptides, with protein abundances being calculated in every run from the sum of all unique normalized peptide ion abundances corresponding to that protein. A protein was considered in the study when present in all six biological replicates in at least one treatment. To assess the effect of excess Mn in the protein profile of tomato xylem sap, we calculated the ratio of normalized protein abundance between treatment and control samples (n = 6). Only changes with a *p* ≤ 0.05 (ANOVA) and a ratio (fold change) ≥2 or ≤0.5 were considered as statistically significant and biologically relevant, respectively. Multivariate statistical analyses (Principal Component Analysis; PCA) were carried out using SPSS Statistical software (v. 24.0), including only proteins showing statistically significant changes (ANOVA; *p* ≤ 0.05) as a result of the excess Mn treatment.

The GO biological process annotation [104] and domain annotations described in the UniProt database were used for classification of each protein identified into one of ten different functional classes: polysaccharide related, oxido-reductases, protein metabolism, carbohydrate metabolism, lipid metabolism, signaling/regulation, defense, nutrient reservoir, unknown and a miscellaneous group containing categories not belonging to the previous groups. The presence of a signal peptide (conventional secretory pathway) was assessed using SignalP (v.5.0) [105], TargetP (v.2.0) [106] and SecretomeP (v.2.0) [107,108]. To assign proteins to unconventional protein secretion (UPS) pathways, three tools were used: SecretomeP2.0, based on mammalian protein data, and the new plant-based tools LSPpred (based on a curated list of likely unconventionally secreted proteins in Arabidopsis) and SLPpred (a SecretomeP-like tool, based on classically secreted proteins, from Arabidopsis with their signal peptide removed) [40,109].

### 4.6. Root Protein Profiling and Comparison with the Xylem Sap

The analysis of root proteins was carried out by shotgun and 2-DE techniques as described in detail in [42]. This combined analysis gave a list of 161 root proteins being reliably identified and quantified with at least two peptides and showing significant changes (ANOVA *p* ≤ 0.05 and fold >2). These proteins were classified in the same categories used for the xylem sap, and both protein databases (xylem sap and roots) were compared to find commonalities.

## 5. Conclusions

The concentration of Mn in the xylem sap increased when compared to that present in the nutrient solution, indicating the presence of an active translocation of this metal from roots to shoots. Excess Mn caused statistically significant and biologically relevant changes in the relative abundance of 322 proteins, with more decreasing than increasing, suggesting that this metal stress causes an overall deactivation of metabolic pathways. Processes most affected by excess Mn were in the oxido-reductase, polysaccharide and protein metabolism classes. Excess Mn induced changes in relative abundance in proteins involved in cell wall degradation and lignin formation, consistent with the existence of alterations in the cell wall. Protein turnover was also affected, as indicated by the decrease in proteolytic enzymes and protein synthesis-related proteins. Excess Mn modified the redox environment of the xylem sap, with changes in the abundance of oxido-reductase and defense protein classes indicating a stress scenario. Results also indicate that excess Mn decreased the amounts of proteins associated with several signaling pathways, including fasciclin-like arabinogalactan-proteins and lipids, as well as those of proteases that may be involved in the release of signaling peptides and protein maturation.

The prediction tools used to identify unconventional secretory proteins gave contrasting results. From a total of 142 proteins, 37 (26%) were predicted as secretory by the bacterial/mammalian protein data-based SecretomeP, but not by the plant data-based tools. This highlights the complexity of the issue and the need to conduct further research towards the development of plant-based tools for predicting protein secretion.

The comparison of the proteins changing in abundance in the roots and xylem sap indicate the response to Mn excess includes both tissue-specific and systemic changes.

## Figures and Tables

**Table 1:** Exudation rates, protein yields, leaf transpiration rates and Mn, Fe, Zn and Cu concentrations in the xylem sap of *Solanum lycopersicum* plants grown in control and excess Mn conditions for 8 days. Data are means ± SE (n = 6 for collection parameters and n = 4 for micronutrient concentrations). Statistically significant differences were calculated with the Student’s *t*-test: * *p* ≤ 0.05; ** *p* ≤ 0.01; *** *p* ≤ 0.001).

*Collection Parameters*	Control	Excess Mn
Exudation rate (mL g^−1^ DW h^−1^)	0.56 ± 0.11	0.52 ± 0.21
Protein yield (ng protein µL^−1^ xylem)	9.0 ± 3.4	27.0 ± 12.5 *
Leaf transpiration rate (mmol H_2_O m^−2^ s^−1^)	10.9 ± 0.5	5.2 ± 0.1 ***
*Micronutrient concentrations (µM)*		
Mn	6.5 ± 0.8	385.7 ± 16.1 ***
Fe	47.8 ± 12.5	15.7 ± 2.5 **
Zn	9.5 ± 1.3	8.0 ± 0.9 *
Cu	3.6 ± 0.8	3.1 ± 0.5

**Table 2:** List of proteins contributing to component 1 in the PCA. Standardized component scores were obtained in the PCA analysis of the differential proteins (ANOVA, *p* ≤ 0.05). Accession and description correspond to identifier and name in the ITAG3.2 database, respectively. Fold-changes (Fold +Mn/Control) were calculated by dividing the mean of normalized abundances (obtained with Progenesis QI v.2.5.1) in plants affected by excess Mn by that of control plants. The single protein with an asterisk after the ITAG database identifier was not included in Table S3 because it was out of the biological threshold range.

Accession	Comp. 1 Score	Comp. 2 Score	Description	Fold +Mn/ Control
*Polysaccharide metabolism (9 proteins)*				
Solyc01g104950.3.1	0.001	0.013	LEXYL2 (GH3)	0.5
Solyc12g056960.2.1	−0.002	−0.061	glucan endo-1,3-β-d-glucosidase (GH17)	4.0
Solyc10g079860.2.1	−0.008	−0.034	glucan endo-1,3-β-d-glucosidase (GH17)	51.6
Solyc01g008620.3.1	−0.024	0.169	glucan endo-1,3-β-glucosidase (GH17)	233.6
Solyc01g059965.1.1	−0.492	−2.371	glucan endo-1,3- β-glucosidase (GH17)	188.7
Solyc02g086700.3.1	−0.001	−0.018	glucan endo-1,3-β-glucosidase-like (GH17)	48.8
Solyc05g050130.3.1	−0.001	0.002	acidic endochitinase (GH18)	18.3
Solyc10g055800.2.1	−0.004	−0.041	endochitinase (GH19)	51.1
Solyc02g082920.3.1	−0.001	0.005	chitinase (GH19)	91.3
*Protein metabolism (5 proteins)*				
Solyc04g078110.1.1	0.002	0.014	serine protease SBT1 (MEROPS peptidase family S8)	0.5
Solyc06g074850.3.1	0.001	0.006	carboxypeptidase (MEROPS peptidase family S10)	0.3
Solyc08g067100.2.1	0.024	0.047	eukaryotic aspartyl protease family protein (MEROPS peptidase family A1)	0.5
Solyc08g079870.3.1	−0.011	0.072	Subtilisin (MEROPS peptidase family S8)	74.3
Solyc08g079900.3.1	−0.390	3.028	subtilisin-like protease (MEROPS peptidase family S8)	57.1
*Oxido-reductases (5 proteins)*				
Solyc06g076630.3.1	0.007	0.093	peroxidase	0.3
Solyc12g094620.2.1	0.015	0.247	catalase	0.2
Solyc01g105070.3.1	−0.012	−0.205	LECEVI16G peroxidase precursor	14.1
Solyc06g005940.3.1	−0.001	−0.001	protein disulfide-isomerase-like	11.5
Solyc09g009390.3.1	−0.001	−0.004	monodehydroascorbate reductase	2.2
*Defense (5 proteins)*				
Solyc01g097240.3.1	−0.003	0.011	pathogenesis-related protein PR-4	153.6
Solyc00g174340.2.1	−0.001	0.003	pathogenesis-related protein PR-1	27.2
Solyc09g090980.3.1	0.003	−0.024	major allergen Mal d 1	25.7
Solyc12g005720.1.1	−0.001	−0.012	cysteine-rich receptor-like kinase protein	9.8
Solyc09g091000.3.1	0.003	−0.016	major allergen d 1	54.3
*Lipid metabolism (2 proteins)*				
Solyc03g079880.3.1	0.003	0.043	protease inhibitor/seed storage/lipid transfer family protein	0.4
Solyc08g067500.1.1	0.002	0.030	non-specific lipid-transfer protein	0.2
*Carbohydrate metabolism (1 protein)*				
Solyc06g073190.3.1 *	0.003	0.66	fructokinase-2	0.7
*Miscellaneous (2 proteins)*				
Solyc01g111170.3.1	0.002	0.032	diageotropica/Peptidyl-prolyl cis-trans isomerase	0.2
Solyc08g074682.1.1	0.001	−0.015	polyphenol oxidase precursor	22.3

* Chloroplastic protein.

**Table 3:** Xylem sap proteins showing large changes in abundance (>25-fold increases or 90% decreases) among the total 263 secretory proteins affected by excess Mn (ANOVA, *p* ≤ 0.05 and fold-change ≥2 or ≤0.5). Accession ITAG3.2 and UniProt indicates the database entry. Abundance changes (change +Mn/Control) were calculated by dividing the mean of normalized abundances (obtained with Progenesis QI v.2.5.1) in plants affected by excess Mn by that in control plants. Description includes the protein name according to the ITAG3.2 and UniProt databases. The column Category indicates the classification (CPS, UPS or sUPS), and the column Prediction tool indicates the algorithms providing true values (SignalP, TargetP, SecretomeP, LSP and SPL). Detailed information about functional classification, identification and quantification is given in Tables S3 and S4.

#	Accession ITAG3.2	Accession UniProt	Change +Mn/Control	Description	Category	Prediction Tool
*Polysaccharide metabolism (13 proteins)*						
1	Solyc09g092170.2.1	A0A3Q7IA20	0.1	β-galactosidase STBG2 (GH35)	CPS	TarP, SecP
2	Solyc07g062210.3.1	A0A3Q7HE74	0.1	protein trichome birefringence-like 41	CPS	SigP, TarP, SecP
3	Solyc04g016470.3.1	A0A3Q7GST1	111.6	glucan endo-1,3-β-D-glucosidase (GH17)	CPS	TarP, SecP
4	Solyc10g079860.2.1	A0A3Q7IKF2	51.6	glucan endo-1,3-β-D-glucosidase (GH17)	CPS	SigP, TarP, SecP
5	Solyc01g008620.3.1	A0A3Q7E938	233.6	glucan endo-1,3-β-glucosidase (GH17)	CPS	SigP, TarP, SecP
6	Solyc01g059965.1.1	Q01413	188.7	glucan endo-1,3-β-glucosidase (GH17)	CPS	SigP, TarP, SecP
7	Solyc02g086700.3.1	A0A3Q7FVX4	48.8	glucan endo-1,3-β-glucosidase-like (GH17)	CPS	TarP, SecP
8	Solyc07g005100.3.1	A0A3Q7H377	30.4	Chitinase/lysozyme (GH18)	CPS	SigP, TarP, SecP
9	Solyc10g055800.2.1	A0A3Q7IHS3	51.1	endochitinase (GH19)	CPS	SigP, TarP, SecP
10	Solyc10g055810.2.1	Q05538	80.9	basic 30 kDa endochitinase (GH19)	CPS	SigP, TarP, SecP
11	Solyc02g082920.3.1	Q05539	91.3	acidic extracellular 26 kD chitinase (GH19)	CPS	SigP, TarP, SecP
12	Solyc10g055820.2.1	A0A3Q7IIQ3	44.4	endochitinase (GH19)	CPS	SigP, TarP, SecP
13	Solyc11g005480.2.1	A0A3Q7IP30	26.1	citrate-binding protein-like	CPS	SigP, TarP, SecP
*Protein metabolism (12 proteins)*						
14	Solyc00g005000.3.1	A0A494G8A2	0.1	eukaryotic aspartyl protease family protein	CPS	SigP, TarP, SecP
15	Solyc07g064590.3.1	A0A3Q7HFD0	0.1	inducible plastid-lipid associated protein	UPS	LSP, SecP
16	Solyc06g008170.3.1	K4CUW3	0.1	50S ribosomal protein L14	sUPS	SecP
17	Solyc09g066430.3.1	A0A3Q7J0J5	0.1	60S ribosomal protein L14	sUPS	SecP
18	Solyc08g079870.3.1	-	74.3	subtilisin (MEROPS S8, clan SB)	CPS	SigP, TarP, SecP
19	Solyc08g079900.3.1	-	57.1	subtilisin-like protease (MEROPS S8, clan SB)	CPS	SigP, TarP, SecP
20	Solyc06g008620.1.1	A0A3Q7GN27	31.7	tolB protein-like protein (MEROPS S9)	UPS	SPL, SecP
21	Solyc01g087850.2.1	O82777	31.0	serine protease SBT3 (MEROPS I13, clan IG)	CPS	SigP, TarP, SecP
22	Solyc03g020010.1.1	O48625	93.3	Miraculin (MEROPS I13, clan IG)	CPS	SigP, TarP, SecP
23	Solyc03g019690.1.1	A0A3Q7FGU5	37.8	kunitz-type protease inhibitor (MEROPS I13, clan IG)	CPS	SigP, TarP, SecP
24	Solyc03g098740.1.1	A0A3Q7FNG4	208.5	kunitz trypsin inhibitor (MEROPS I3, clan IC)	CPS	SigP, TarP, SecP
25	Solyc08g080630.3.1	A0A3Q7IQ00	31.8	ethylene-responsive proteinase inhibitor 1 (MEROPS I3, clan IC)	CPS	SigP, TarP, SecP
*Oxido-reductases (6 proteins)*						
26	Solyc06g005160.3.1	Q3I5C4	0.1	Ascorbate peroxidase	UPS	LSP
27	Solyc04g074740.3.1	A0A3Q7G526	0.0	blue copper protein-like	CPS	SigP, TarP, SecP
28	Solyc08g066740.3.1	A0A3Q7HR33	0.1	early nodulin-like protein 1-like	CPS	SigP, TarP, SecP
29	Solyc08g028690.3.1	A0A3Q7HMQ9	0.0	NAD(P)-binding Rossmann-fold superfamily protein	UPS	LSP, SPL, SecP
30	Solyc03g006700.3.1	A0A3Q7FFR5	28.7	peroxidase (AtPrx52)	CPS	SigP, TarP, SecP
31	Solyc04g071890.3.1	-	71.6	peroxidase	CPS	TarP, SecP
*Defense (5 proteins)*						
32	Solyc01g097240.3.1	P32045	153.6	pathogenesis-related protein PR-4	CPS	SigP, TarP, SecP
33	Solyc00g174340.2.1	A0A494GA45	27.2	pathogenesis-related protein 1	CPS	SigP, TarP, SecP
34	Solyc01g106620.2.1	B2LW68	61.8	pathogenesis-related protein 1	CPS	SigP, TarP, SecP
35	Solyc07g005380.3.1	A0A3Q7H2K6	37.7	pathogenesis-related PR-10-related/norcoclaurine synthase-like protein	UPS	SPL, SecP
36	Solyc09g090980.3.1	-	25.7	major allergen Mal d 1	UPS	SPL
*Carbohydrate metabolism (4 proteins)*						
37	Solyc01g094200.3.1	A0A3Q7EKQ6	0.1	malic enzyme	UPS	LSP, SecP
38	Solyc01g101040.3.1	A0A3Q7EPC2	0.1	ATP-citrate synthase	UPS	LSP
39	Solyc01g058390.3.1	A0A3Q7EEN4	0.1	galactokinase	sUPS	SecP
40	Solyc02g088690.3.1	A0A3Q7F9B8	0.1	UDP-glucose 6-dehydrogenase family protein	UPS	LSP, SecP
*Signaling/regulation (5 proteins)*						
41	Solyc02g063090.3.1	A0A3Q7FJZ3	0.1	T-complex protein 1 subunit zeta 1	UPS	LSP
42	Solyc05g056310.3.1	A0A3Q7GLM4	0.1	T-complex protein 1 subunit gamma	UPS	LSP
43	Solyc01g086920.3.1	A0A3Q7F2I5	0.0	leucine-rich repeat receptor-like protein kinase family	CPS	SigP, TarP, SecP
44	Solyc10g050110.1.1	A0A3Q7IFT3	0.1	leucine-rich repeat receptor-like protein kinase family	CPS	SigP, TarP, SecP
45	Solyc08g082820.3.1	A0A3Q7HX02	31.3	tomato BiP (binding protein)/grp78 (HSP70)	CPS	SigP, TarP, SecP
*Lipid metabolism (1 protein)*						
46	Solyc01g107990.3.1	A0A3Q7FCU3	0.1	PI-PLC X domain-containing protein	CPS	SigP, TarP, SecP
*Miscellaneous (4 proteins)*						
47	Solyc03g115630.3.1	A0A3Q7FR13	0.1	carbamoyl-phosphate synthase	UPS	LSP, SPL, SecP
48	Solyc06g005360.3.1	A0A3Q7GNQ9	0.1	actin-depolymerizing factor family protein	sUPS	SecP
49	Solyc07g064160.3.1	A0A3Q7HFB9	0.1	thiamine thiazole synthase	sUP	SecP
50	Solyc10g049970.2.1	A0A3Q7IFZ6	24.9	kynurenine formamidase	CPS	SigP, TarP, SecP
*Unknown function (1 protein)*						
51	Solyc06g035920.3.1	A0A3Q7GU06	0.1	remorin	sUPS	SecP

**Table 4:** Comparison of the changes in the xylem and root protein profiles. List of proteins showing statistically significant (ANOVA, *p* ≤ 0.05) and biologically relevant (fold ≥ 2 or ≤ 0.5) changes in abundance in both root and xylem sap proteomes when tomato plants grown in excess Mn were compared to controls. The column Category indicates the classification (CPS, UPS or sUPS), and the column Prediction tool indicates the algorithms providing true values (SignalP, TargetP, SecretomeP, LSP and SPL). Proteins changing in abundance in roots as a result of excess Mn were described in [42].

					Xylem sap	Root
			Category	Prediction Tool	Fold +Mn/Control	Fold +Mn/Control
*Proteins decreasing in both proteomes (16 proteins)*						
Polysaccharide metabolism	Solyc01g104950.3.1	β -D-xylosidase 2 precursor (LEXYL2)	CPS	SigP, TarP, SecP	0.5	0.4
	Solyc05g012070.3.1	UDP-glucose:protein transglucosylase-like protein	UPS	SPL	0.2	0.5
Protein metabolism	Solyc02g068740.3.1	glycine cleavage system H family protein (MEROPS peptidase family C1)	CPS	SecP	0.3	0.5
	Solyc12g008630.2.1	mitochondrial-processing peptidase subunit α-like (MEROPS peptidase family M16)	UPS	LSP, SecP	0.4	0.3
	Solyc02g081700.1.1	proteasome subunit α type (MEROPS peptidase family T1A)	UPS	LSP, SecP	0.5	0.4
	Solyc06g073310.3.1	60S ribosomal protein l9	sUPS	SecP	0.5	0.5
Oxido-reductases	Solyc06g005160.3.1	ascorbate peroxidase	UPS	LSP	0.1	0.2
	Solyc01g107590.3.1	cinnamyl alcohol dehydrogenase	UPS	LSP	0.2	0.3
Amino acid metabolism	Solyc02g080810.3.1	aminomethyltransferase	UPS	LSP, SecP	0.4	0.5
	Solyc01g080280.3.1 *	glutamine synthetase	lnS	LSP	0.4	0.5
Carbohydrate metabolism	Solyc08g080140.3.1	3,5-epimerase/4-reductase	sUPS	SecP	0.3	0.2
Signaling/regulation	Solyc06g065520.3.1	T-complex protein 1 subunit eta	UPS	LSP	0.3	0.2
Miscellaneous	Solyc02g087300.1.1	transducin/WD40 repeat-like superfamily protein	UPS	SPL, SecP	0.4	0.5
	Solyc01g006280.3.1	formate-tetrahydrofolate ligase	UPS	LSP, SPL	0.4	0.3
	Solyc01g109660.2.1	meloidogyne-induced giant cell protein DB275 (glycine-rich RNA-binding protein)	lnS	None	0.3	0.5
	Solyc12g098150.2.1	Aldo/keto reductase	lnS	None	0.2	0.5
*Proteins increasing in both proteomes (12 proteins)*						
Polysaccharide metabolism	Solyc01g059965.1.1	β-1,3-glucanase (GH17)	CPS	SigP, TarP, SecP	188.7	8.3
	Solyc07g005100.3.1	chitinase/lysozyme (GH18)	CPS	SigP, TarP, SecP	30.4	3.4
	Solyc10g055800.2.1	chitinase (GH19)	CPS	SigP, TarP, SecP	51.1	3.6
	Solyc10g055810.2.1	chitinase Z15140 (GH19)	CPS	SigP, TarP, SecP	80.9	4.9
	Solyc10g055820.2.1	chitinase (GH19)	CPS	SigP, TarP, SecP	44.4	9.5
Protein metabolism	Solyc03g019690.1.1	Kunitz-type protease inhibitor (MEROPS I3, clan IC)	CPS	SigP, TarP, SecP	37.8	105.3
Oxido-reductases	Solyc03g006700.3.1	peroxidase	CPS	SigP, TarP, SecP	28.7	8.4
Defense	Solyc01g097240.3.1	pathogenesis-related protein PR-4	CPS	SigP, TarP, SecP	153.6	12.2
	Solyc01g106620.2.1	pathogenesis-related protein 1	CPS	SigP, TarP, SecP	61.8	4.3
	Solyc09g091000.3.1	Major allergen d 1	lnS	None	54.3	10.5
Miscellaneous	Solyc10g049970.2.1	kynurenine formamidase	CPS	SigP, TarP, SecP	24.9	32.6
	Solyc09g090430.3.1	cyanate hydratase	lnS	None	3.4	2.4
*Proteins with opposite responses in root and xylem sap proteomes (2 proteins)*						
Lipid metabolism	Solyc12g017460.1.1	GDSL esterase/lipase At1g28590-like	CPS	SigP, TarP, SecP	13.5	0.5
Signaling/regulation	Solyc01g106210.3.1	heat shock protein 70	UPS	LSP	3.4	0.4

* Chloroplastic protein.

## Supplementary Materials

The following are available online at https://www.mdpi.com/1422-0067/21/22/8863/s1, Figure S1: Effect of excess Mn on the xylem sap protein profile using label-free shotgun proteomic analyses, showing the different protein categories. Figure S2: UpSet plot showing the number of proteins lacking a signal peptide, but predicted to be secretory by the three different prediction tools used in the study. Table S1. List of proteins detected in tomato xylem sap by shotgun proteomics and Progenesis LC-MS analyses. Table S2. List of proteins contributing to component 1 and component 2 in the PCA. Standardized component scores were obtained in the PCA analysis of the differential proteins (ANOVA, *p* ≤ 0.05). Table S3. List of proteins that presented significant decreases (ANOVA, *p* ≤ 0.05; quantification with at least 2 peptides; fold ≤0.5) when xylem sap samples of plants grown in control conditions were compared to those grown in Mn toxicity by shotgun proteomics. Table S4. List of proteins that presented significant increases (ANOVA, *p* ≤ 0.05; quantification with at least 2 peptides; fold ≥2) when xylem sap samples of plants grown in control conditions were compared to those grown in Mn excess by shotgun proteomics. Table S5. List of proteins that presented increases between 8,33-25 (ANOVA, *p* ≤ 0.05; quantification with at least 2 peptides; fold ≥2) when xylem sap samples of plants grown in control conditions were compared to those grown in Mn-excess by shotgun proteomics.
