# Supplementary material for: Effects of Excess Manganese on the Xylem Sap Protein Profile of Tomato (Solanum lycopersicum) as Revealed by Shotgun Proteomic Analysis
Source: Int J Mol Sci. 2020 Nov 23;21(22):8863. doi: 10.3390/ijms21228863 (PMC7700171; doi:10.3390/ijms21228863)
Supplement: Supplementary file 1 [file ijms-21-08863-s001.zip › ijms-984506 Supplementary files/ijms-984506 Suppl Figs S1-S2.pdf]

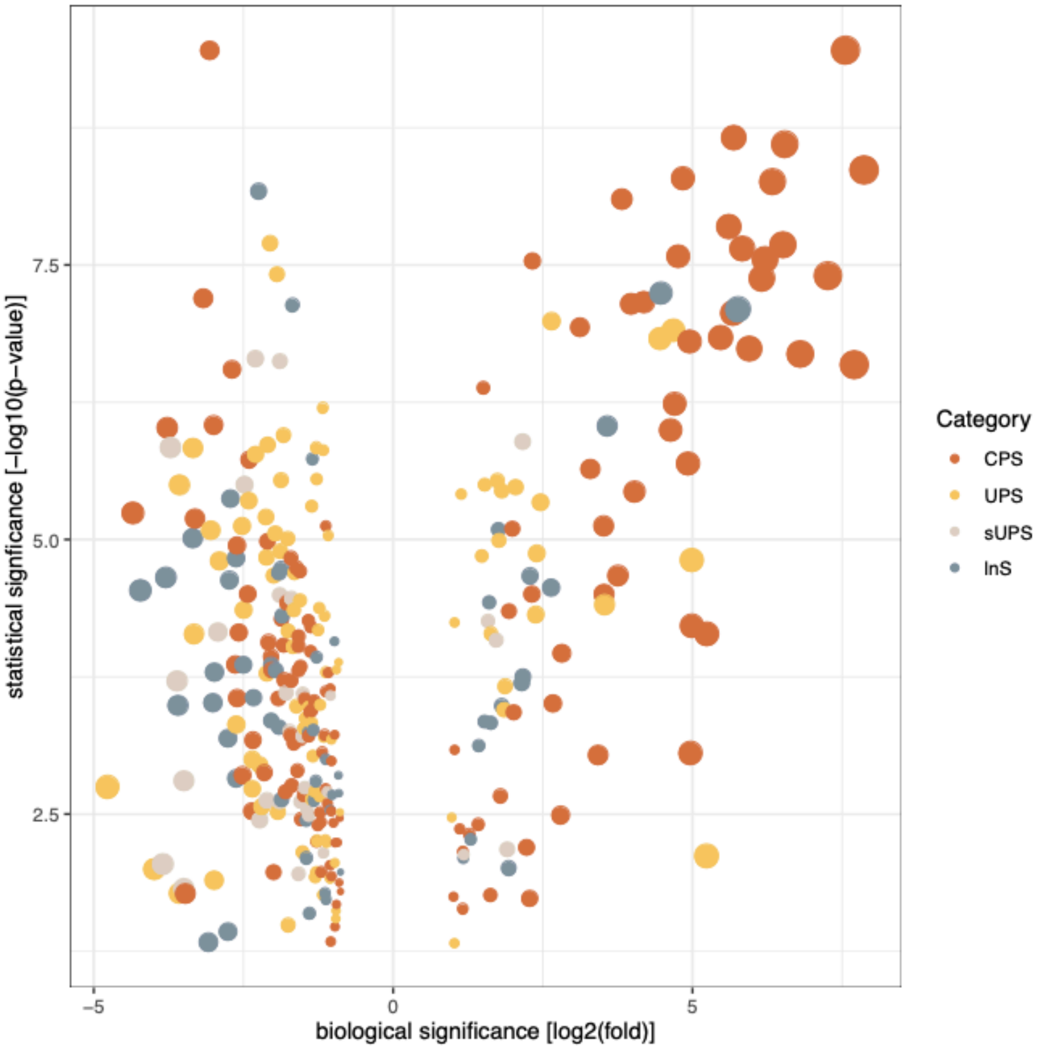

Figure S1 Effect of excess Mn on the xylem sap protein profile using label-free shotgun proteomic analyses, showing the different protein categories. The volcano scatter plot contains the 668 identified and quantified proteins (peptides assigned to a protein and used for quantification ≥ 2). CPS: conventional secretory pathway; UPS: unconventional secretory pathway; sUPS: suggested unconventional secretory pathway; lnS: likely non-secretory.

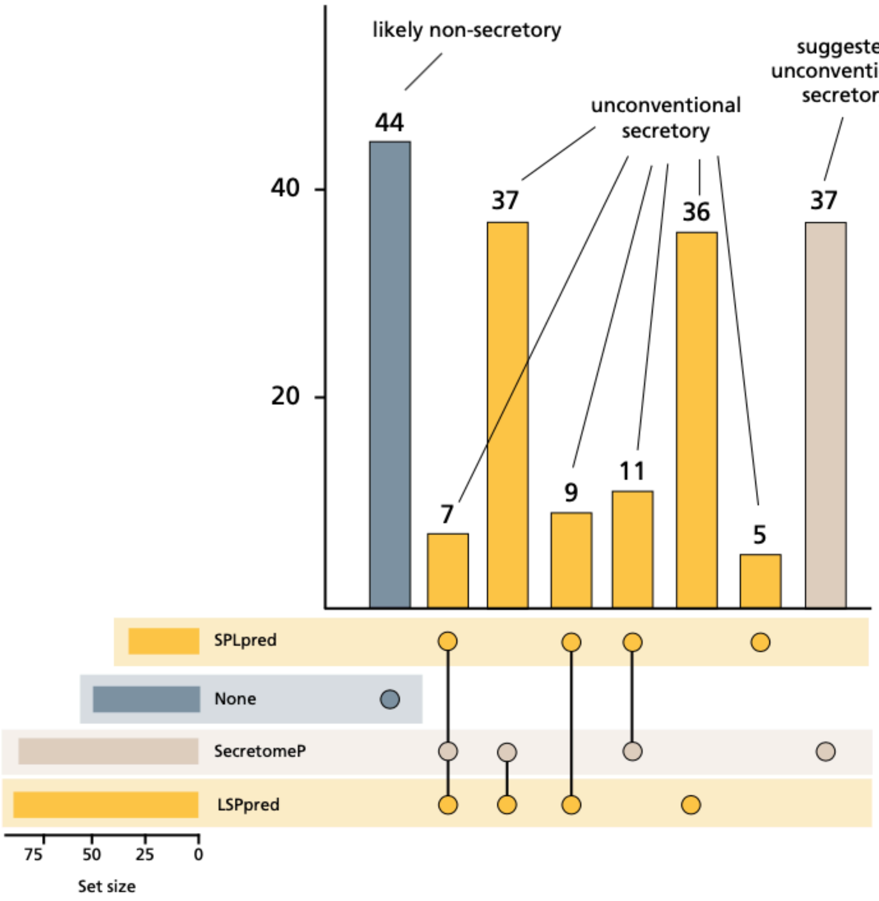

Figure S2 UpSet plot showing the number of proteins lacking a signal peptide, but predicted to be secretory by the three different prediction tools used in the study. Those predicted to be secretory by LSPpred and/or SPLpred were considered `unconventional secretory´; those predicted to be secretory by Secretome P only were considered `suggested unconventional secretory', and those not considered to be secretory by any tool were considered `likely non-secretory'.
